# Epigenomic Dysregulation in Schizophrenia: In Search of Disease Etiology and Biomarkers

**DOI:** 10.3390/cells9081837

**Published:** 2020-08-05

**Authors:** Behnaz Khavari, Murray J. Cairns

**Affiliations:** 1 School of Biomedical Sciences and Pharmacy, University of Newcastle, Callaghan, NSW 2308, Australia; behnaz.khavari@uon.edu.au; 2 Centre for Brain and Mental Health Research, University of Newcastle and the Hunter Medical Research Institute, Newcastle, NSW 2308, Australia

**Keywords:** DNA methylation, histone modification, miRNA, long non-coding RNA, Schizophrenia

## Abstract

Schizophrenia is a severe psychiatric disorder with a complex array of signs and symptoms that causes very significant disability in young people. While schizophrenia has a strong genetic component, with heritability around 80%, there is also a very significant range of environmental exposures and stressors that have been implicated in disease development and neuropathology, such as maternal immune infection, obstetric complications, childhood trauma and cannabis exposure. It is postulated that epigenetic factors, as well as regulatory non-coding RNAs, mediate the effects of these environmental stressors. In this review, we explore the most well-known epigenetic marks, including DNA methylation and histone modification, along with emerging RNA mediators of epigenomic state, including miRNAs and lncRNAs, and discuss their collective potential for involvement in the pathophysiology of schizophrenia implicated through the postmortem analysis of brain tissue. Given that peripheral tissues, such as blood, saliva, and olfactory epithelium have the same genetic composition and are exposed to many of the same environmental exposures, we also examine some studies supporting the application of peripheral tissues for epigenomic biomarker discovery in schizophrenia. Finally, we provide some perspective on how these biomarkers may be utilized to capture a signature of past events that informs future treatment.

## 1. Introduction

Schizophrenia (SZ) is a debilitating psychiatric disorder that affects 0.7% of people at some point in their life [1]. It usually occurs early in adolescence or adulthood and persists for most of an individual’s life and is recognized as one of the top 15 leading causes of disability worldwide [2]. The disorder is characterized by: (1) positive symptoms, including delusions as well as visual, auditory, olfactory, and tactile hallucinations; (2) negative symptoms, such as social withdrawal, anhedonia, loss of affect, including monotonous voice tone and immobile facial expressions; (3) cognitive signs, such as impairments in attention, learning, problem solving, and working memory [3].

The incidence of schizophrenia is known to be higher among relatives of affected individuals compared to the general population. For example, children with one affected parent have a 17% chance of developing schizophrenia in their lifetime, and this increases to 35% when both parents are affected [4]. The high heritability of disease has prompted the very substantial investigation of genetic factors through linkage studies, candidate gene studies, and more recently, genome-wide association studies (GWAS), with the hope of identifying a molecular basis for the disorder. While several family-based studies identified rare variations associated with disease development, they only contribute a small fraction of the overall population risk for this relatively common disorder. Large GWAS of schizophrenia have been more informative with the discovery of more than 140 loci [5,6]. The investigation of probe intensity values (Log R ratio and B-allele frequency) of this genome-wide date, also identified several rare disease-associated structural variants with larger effect size [7]. Collectively these large GWAS highlight the genomic complexity of the syndrome with most of the risk being distributed across a large number of small effect size variants, which are highly heterogeneous between individuals.

Despite the high heritability rate, estimated to be around 80%, several environmental factors have also been suggested to contribute to the development of schizophrenia. These include maternal immune activation, hypoxia, nutrient deprivation, maternal deprivation, and various toxins. Based on the results from animal studies, it has been speculated that many of these environmental exposures have a lasting impact through epigenetic modifications that accumulate in the brain and other tissues throughout development and alter the function of the genome. The role of these exposures and their interaction with genomic sequence variation, is consistent with the neurodevelopmental hypothesis of schizophrenia, which suggests that schizophrenia arises from a heritable risk and/or environmental exposures that occur throughout development, particularly prenatally, as well as early in life and during adolescence [8,9,10].

While postmortem investigations of disease-associated epigenomic changes in the brain of individuals with a diagnosis of schizophrenia have provided valuable information about the disease etiology, and supported established hypotheses about the etiology, there are several limitations. Firstly, there are several confounding factors affecting the epigenome of the postmortem brain, such as age, sex, cause of death, tissue PH, postmortem interval (PMI), and medication history [11]. Secondly, it should be always taken into account that the observed modifications in the epigenome might simply be the consequence of the disease state, not necessarily the underlying cause of it. Thirdly, due to the relatively low number of donors, studies are typically conducted on small sample numbers with limited statistical power. While these observations are useful for discovering disease etiology and insights for the development of new drugs, the analysis of the postmortem brain does not necessarily help to identify biomarkers for early disease diagnosis.

As an alternative to studies focusing on the central nervous system, epigenetic dysregulation in peripheral tissues, such as blood, serum, saliva, and olfactory epithelium, which are more accessible in living patients, have been widely investigated. While these studies are useful for detecting biomarkers, they can also provide additional insight into disease etiology with some concordance reported between gene expression and epigenomic modification in blood and brain tissue. Moreover, peripheral tissues may be particularly important biomarkers of many of the environmental risk factors associated with schizophrenia, such as stress and other exposures, including hormones, hypoxia, nutrient deprivation, and proinflammatory cytokines.

In this review, we examine some of the current literature exploring changes in DNA methylation, histone modifications, and RNA mediators of epigenomic state, including microRNAs (miRNAs) and long non-coding RNAs (lncRNAs), in the brain and peripheral tissue in schizophrenia patients and discuss their involvement in disease etiology and potential application as state and trait biomarkers of environmental exposures. During the preparation of this manuscript, two systematic reviews with similar objectives were published, although these did not consider long non-coding RNA [12,13].

## 2. Epigenetics in Brain, Behavior, and the Neuropathology of Schizophrenia

The brain can be visualized as a higher order epigenetic device that senses environmental stimuli, records it, and processes information to direct motivational activity that organizes orientation of the organism towards survival and reproduction. In large complex brains, this is achieved through the regulation of neural circuits, but at the heart of each node there is a cell that responds to inputs by recording its history of activation or inactivation through epigenomic changes, which, in turn help to regulate its structural and functional connectivity in the network. When we think about the brain in this way, it is reasonable to suggest that epigenetic mechanisms are a central feature of this extraordinary organ. However, as with any complex regulated system, there is tremendous opportunity for systematic failure, which can lead to a breakdown in complexity and a reduction in structure and function. In the human brain, this may contribute to cognitive and behavioral syndromes, such as schizophrenia. For example, we know from a wealth of imaging [14], neurophysiology [15], and neuropathological examination [16] that this disorder is associated with a loss of structural and functional connectivity [17], which could be derived to some extent by epigenomic mechanisms that fail to integrate environmental input received through neural activity or other signaling mechanisms. This is supported through several lines of evidence from the genetics, through cell biology, to molecular neuropathology. This research has focused on the central mechanisms for epigenomic modification that have been established for several decades, such as DNA and histone modification—highlighted below—that alter the accessibility of chromatin as well as more nuanced interactions that alter gene expression. Other important aspects of epigenomic regulation that have emerged more recently are those contributed by non-coding RNAs. These structurally and functionally diverse molecules help shape epigenomic transitions, as well as directly modulating expression state in response to genomic and environmental signals. Evidence for the involvement of these dynamic nucleic acids in schizophrenia and other psychiatric syndromes has also been emerging, which collectively weaves a rich tapestry for molecular dysfunction in epigenomic regulation, expressed through the disruption of neural connectivity and homeostasis.

### 2.1. DNA Methylation

DNA methylation is the most stable and well-characterized epigenetic modification, which mainly occurs in cytosine and guanine-rich segments of the genome known as CpG islands. These are typically regions of more than 500 base pairs with a GC content larger than 55% in the form of CpG dinucleotide clusters. The process is catalyzed and maintained by a family of DNA methyltransferases (DNMTs), including DNMT1, DNMT3A and DNMT3B, and involves the addition of a methyl group to the C5 position of cytosine at a CpG dinucleotide. Recent studies have revealed that DNA methylation can also occur at sites other than CpG sequences. This non-CpG methylation is found at cytosines, followed by thymine, adenine, or another cytosine, and is catalyzed by DNMT3A and DNMT3B, and is suggested to be prevalent in human embryonic stem cells and brain tissue. However, their exact functions and mechanisms are, as yet, poorly understood. Interestingly, unlike CpG methylation, which happens during early development and remains constant over time, non-CpG methylation occurs during hippocampus postnatal development and its level increases over time. While non-CpG methylation was observed to be relatively sparse in the human fetal frontal cortex, it dramatically increases during development in accordance with synaptogenesis and increased synaptic density [18,19]. In most cases, but not exclusively, DNA methylation results in a decrease in gene expression [20].

### 2.2. Disruption of DNA Methylation in Postmortem Brain

DNA methylation changes in schizophrenia have been explored using candidate genes strategy and whole genome approaches. One of the most studied candidate genes displaying differential methylation in schizophrenia is reelin (RELN). Reelin has a crucial role in the extension of axons and dendrites [21] as well as specifying the six-layer structure of the human cortex [22]. More recently, it has been shown to participate in neuronal migration during early postnatal development [23]. Genetic studies have also suggested an association between RELN variants and schizophrenia [24,25]. RELN expression has been observed to be reduced between 30–50% in different areas of the brain in individuals with schizophrenia compared to healthy controls [26,27], which is highly significant given its important role in brain development and connectivity. Through the application of bisulfite modified DNA sequencing and methylation specific PCR (MSP), Abdolmaleky et al. [28] were the first group who attributed RELN expression reduction to the hypermethylation of its promoter in five postmortem frontal lobe brain samples from male patients with schizophrenia compared to healthy individuals. Although similar results were reported by Grayson et al. [29], two later studies did not observe changes in the methylation status of the RELN promoter [30,31]. It was argued that the contradiction was due to differences in experimental protocols and in the brain regions explored [30]. In mice, RELN promoter hypermethylation resulted in the downregulation of Reelin and the presentation of schizophrenia-associated behavioral modifications in response to prenatal restraint stress, indicating that environmental stressors can cause brain dysfunction through modifying the DNA methylation of the RELN gene [32].

Glutamic acid decarboxylase 1 or GAD1 (GAD67) is another gene that has been repeatedly observed to be downregulated in postmortem analysis of the cerebral cortex in schizophrenia [33], which is shown to be associated with its promoter hypermethylation, reviewed by Guidotti et al. [34]. Looking at the results of animal studies, we speculate this decrease could be caused by exposure to schizophrenia-associated environmental risk factors, as a mouse study revealed that maternal immune activation increases Gad1 promoter methylation, which is accompanied by reduced mRNA expression [35]. These genes (including RELN) play a central role in the regulation of GABAergic neurotransmission, which is thought to be a significant component of the neuropathology of schizophrenia [36].

Similarly, COMT is a gene of interest in methylation studies of psychosis because its product, catechol-O-methyltransferase, is involved in the metabolism of dopamine, which is a major driver of positive symptoms and supports the dopamine hypothesis of schizophrenia [37]. Copy number variation (CNV) in the 22q11.2 region, which encompasses the gene, also showed the strongest association with schizophrenia in the largest GWAS of structural variations [7]. COMT promoter methylation studies in 115 postmortem brain samples from the frontal lobe of schizophrenia patients, revealed that the promoter of the gene encoding the membrane-bound isoform is frequently hypomethylated [38]. In contrast, no schizophrenia-associated changes were observed in the frontal cortex in a cohort of 35 schizophrenia cases and 35 matched controls [31]. Another gene, SOX10, which encodes a crucial transcription factor for oligodendrocyte differentiation, has also been shown to have reduced expression in postmortem schizophrenia patients that was correlated with hypermethylation [39]. Moreover, there are several other genes, such as NR3B, GRIA2, and FOXP2, whose expression alterations in schizophrenia have been explored in terms of the involvement of changes in DNA methylation status, and were discussed in detail previously [37] (see summary of mentioned candidate gene methylation studies in Table 1). All in all, these studies suggest that schizophrenia-associated changes in cortical gene expression, including the well-known candidates, RELN and GAD67, might be due to changes in their promoter methylation status—possibly in response to exposure to environmental risk factors for psychiatric disease.

In addition, it has been suggested that changes in DNA methylation in schizophrenia may be more widespread than expected, supporting the application of genome-wide analyses of differential methylation. The advance of technology has provided an opportunity for genome-wide methylation studies, called epigenome-wide association studies (EWAS), through high-throughput microarray- and sequencing-based methods. Examples of hybridization-based technologies include CHARM (Comprehensive High-Throughput Relative Methylation) which uses restriction enzymes sensitive to methylation, and Infinium Assay, which is based on the chemical conversion of DNA by bisulfite treatment. Similarly, the sequencing-based technologies, BS-seq and RRBS (Reduced Representation Bisulfite Sequencing), use the chemical conversion of DNA [40]. A whole-genome methylation study on the frontal cortex from 35 schizophrenia cases and 35 healthy controls reported schizophrenia-associated differences in DNA methylation in a plethora of loci, including many genes functionally related to disease etiology, such as the glutamate-receptor genes NR3B (GRIN3B) and GRIA2, hypo-methylated specifically in male patients, and also genes involved in GABAergic neurotransmission pathways, such as MARLIN-1 (JAKMIP1) and KCNJ6, which are hyper-methylated in females and males, respectively [31]. In a genome-wide DNA methylation study on the frontal cortexes of samples from a cohort of 24 schizophrenia patients and 24 unaffected controls, DNA methylation status were assessed at more than 485,000 CpG sites using the Illumina Infinium HumanMethylation450K Bead Chip, which showed significant differential methylation between the two groups, including 47.3% hypomethylation in the schizophrenia group. Some of the genes with differential methylation were previously reported to be associated with schizophrenia, such as AKT1, NOS1, DNMT1, PPP3CC, DTNBP1, and SOX10 [41]. Interestingly, 99 of the differentially methylated genes were previously reported to be differentially methylated in the peripheral blood cells of schizophrenia subjects [42], confirming the concordance between the brain and blood in terms of epigenetic modifications.

### 2.3. Histone Modifications

Histone modifications occur in four forms: histone acetylation, deacetylation, methylation, and phosphorylation. Histone acetyltransferase (HAT) acetylates lysine residues on the N-terminus of histones 3 and 4 of nucleosomes and gives rise to an increase in gene expression. Conversely, histone deacetylation by histone deacetylase (HDAC) results in less accessibility of promoters to transcription machinery, reducing gene expression. Histone methylation, catalyzed by polycomb repressive complex 2 (PRC2), can induce both the silencing or elevation of gene expression, with the methylation of lysine 9 (H3K9) or 27 (H3K27) on histone 3, resulting in gene suppression, while the methylation of the fourth lysine on histone 3 (H3K4) gives rise to an increase in gene expression. The phosphorylation of serine 10 of histone H3 is another transcription-activating modification which is catalyzed by the Mediator complex [20,43,44,45].

### 2.4. Histone Modification Studies in Postmortem Brain

Several layers of evidence suggest that there is histone modification associated with the development of schizophrenia. These include changes in the expression of several histone modifying enzymes, such as reduced expression of HDAC2 in the dorsolateral prefrontal cortex of schizophrenia cases [46], and increased expression of HDAC1 in the prefrontal cortex of patients [47]. Several genetic variants in histone modifying enzymes have also been associated with the disease, such as the SNP rs1063639 in the histone deacetylase 4 (HDAC4) enzyme in a Korean population [48]. Additionally, pathway analysis in a genome-wide association study of more than 60,000 cases with schizophrenia, bipolar disorder, and depression showed the strongest association for histone methylation processes [49].

To date, little is known about histone modifications in the context of schizophrenia and this could be due to the technical difficulties associated with postmortem analyses of these marks. Despite these challenges, Tang et al. applied ChIP assay to postmortem human prefrontal cortex using an antibody against acetylated histone 3 lysines 9 and 14 (ac-H3K9K14). They then used quantitative PCR with primers against gene promoters, and reported the correlation of gene expression levels with changes in histone H3 acetylation at promoters of four schizophrenia-related genes, including GAD67, translocase of outer mitochondrial membrane 70 homolog A (TOMM70A), 5-hydroxytryptamine receptor 2C (HTR2C), and protein phosphatase 1E (PPM1E) [50]. In another study, the decreased expression of the metabolic genes CRYM, CYTOC/CYC1, MDH, and OAT in the prefrontal cortex of a subset of schizophrenia patients was attributed to high levels of H3-(methyl)arginine 17 (H3meR17) [51]. Applying chromatin immunoprecipitation, Huang et al. observed a decrease in the H3K4-trimethylation (H3K4me3) level in the promoter of the GAD67 gene, which was accompanied by a decrease in its expression, predominantly in female patients, while the maturation of the human prefrontal cortex was associated with increased H3K4me3 and a resultant increase in the GAD67 expression and other GABAergic genes, such as GAD2, NPY, and SS [52]. The decrease in the expression of GAD67 was also reported in a microarray study of the National Brain Bank cohort (16 schizophrenia patients and 27 controls) and shown to be negatively correlated with an increase in the expression of the histone deacetylase enzyme HDA1 [47]. Collectively, these studies, along with several reports of alterations in patients’ cortical levels of histone modifying enzymes, and the observation that therapeutic doses of the mood stabilizer valproate act as a histone deacetylase (HDAC) inhibitor in schizophrenia cases [12], suggest that risk factors for schizophrenia could impact their deteriorating effects on the brain through histone modifications that influence the transcriptional state of genes, such as GAD67, associated with the disorder.

## 3. RNA Mediators of Epigenomic State

### 3.1. MicroRNA (miRNA)

**Figure 1:**
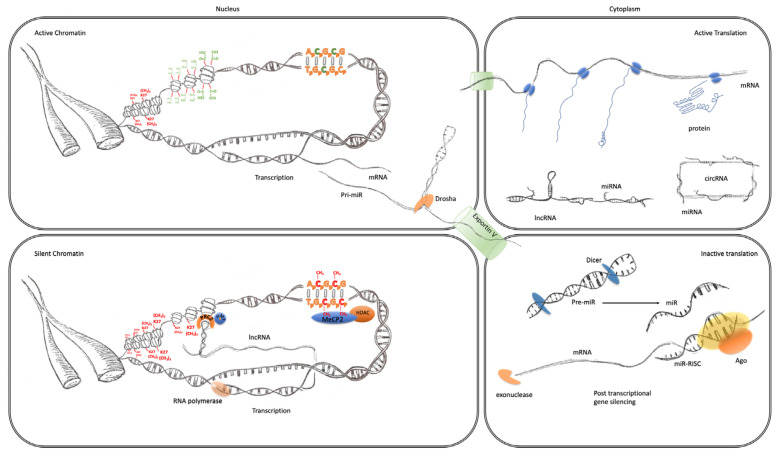
Transcriptional and post-transcriptional regulation of gene expression. The four panels of this schematic are representative of the major epigenomic mechanisms’ associated active and inactive states in the nucleus and cytoplasm. Active chromatin (top left) characterized by an expanded or open euchromatin domain is established by the depletion of histone H3 lysing 27 trimethylation (K27(CH3)3 red) and an increase in histone H3 and H4 acetylation (green). This gives rise to mRNA transcription and non-coding RNA, such as primary miRNA transcripts. After interaction with the microprocessor complex (Drosha/DGCR8), the processed pre-miR are exported to the cytoplasm via exportin 5. mRNA is also exported to the cytoplasm (top right panel) where it becomes associated with ribosomes and translated into protein (blue). This is facilitated by the suppression of potentially active miRNA, which are sequestered through interaction with lincRNA and circRNA. Inactive chromatin (bottom left) is associated with cytosine methylation (red), which is particularly important at CpG islands and promoters where they can bind to MeCP2 which can recruit histone de-acetylases (HDAC). These reduce histone acetylation, which leads to greater compaction of chromatin into a state known as heterochromatin. LincRNA transcripts can also form a scaffold for the assembly of a ribonucleoprotein complex, including the PRC2, that directs the activity of histone methyltransferases (HMTs) that catalyze the trimethylation of histone H3 lysine 27. This modification also further enhances the contraction of chromatin into inactive heterochromatin. Post-transcriptional regulation of gene expression (bottom right panel) can also be facilitated by the maturation of pre-miRs through the activity of dicer. The mature miRNAs guide the RNA-induced silencing complex (comprising Argonaute proteins and other co-factors) to the 3′UTR of their cognate mRNA, causing translational repression and degradation through exonuclease activity.

MicroRNA (miRNA) are a class of small non-coding RNA which are approximately 22 nucleotides of length and transcribed from different regions of the genome, including the exons and introns of host genes, as well as intergenic segments. Their biogenesis is usually initiated by the transcription of the miRNA gene by RNA-polymerase II to produce primary miRNA (pri-miRNA). In the nucleus, pri-miRNA is processed by the microprocessor complex, consisting of Drosha and DGCR8 (DiGeorge syndrome critical region 8), into a 70-nucleotide stem-loop or hairpin structure, called pre-miRNA. Exportin 5 and Ran-GTP transport pre-miRNA into the cytoplasm where it is further processed by the endonuclease Dicer to give rise to a mature double-stranded miRNA ~22 nucleotides long. The last step is loading one of these strands into the RNA-induced silencing complex (RISC), containing Dicer, TAR RNA binding protein, and a member of the Argonaute family. Through their 5′-end seed region, comprising nucleotides 2–8, miRNA can bind to a target sequence on the 3′-UTR of mature mRNAs, which is called the miRNA response element (MRE), and lead to either the degradation of mRNA or repression of its translation (Figure 1). As each miRNA needs only partial homology/complementarity to their target mRNAs, to achieve post-transcriptional silencing, they can each affect the expression of hundreds of genes simultaneously and synchronize multiple components of independent signaling pathways [53].

While miRNAs are generally known as cytoplasmic repressors of gene expression at post-transcriptional level, emerging advances from virus [54] and human [55,56,57,58,59,60,61,62] studies show they are also enriched in the nucleus [61,62,63] and able to repress [58,59,60,62] and even activate [55,56,57,64] gene expression at transcription level through directly binding to complementary sequences in the genome at gene promoters [55,56,57,58,59,60,62,64] and enhancers [65]. These observations, alongside several reports of the miRNA-directed repression of epigenetic enzymes, such as the DNA methyltransferases DNMT1, DNMT3A and DNMT3B [66,67], the histone methyltransferase EZH2 [68], and the DNA methyltransferase DNMT3b [69], indicate that miRNAs are epigenetic machinery factors [58,60,70,71].

### 3.2. Postmortem Investigation of miRNA in Schizophrenia

miRNAs are believed to have a crucial role in the development of central nervous system. This was exemplified in a study by Miller et al., which reported that the upregulation of miR-132 in the mouse led to the downregulation of its target genes during brain development [72]. For a detailed review see Rajman and Schratt [73]. The differential expression of miRNAs has also been reported in schizophrenia and may be an important mechanism influencing the development of the disorder, which is associated with substantial dysregulation of gene expression. We hypothesized that, in many cases, this alteration could be part of the response to environmental exposures, such as inflammatory stress. To address this hypothesis, we treated pregnant mice with poly I:C, a potent inducer of maternal immune activation, and observed significant differences in miRNA expression in the entorhinal cortex (EC) of the offspring’s left hemisphere [74]. This model was previously shown to produce behavioral deficits similar to those observed in patients with schizophrenia, including excessive behavioral switching and impaired working memory [75].

Several investigations have now observed changes in the miRNA expression profile in the postmortem brains of subjects with schizophrenia. Perkins et al. reported that 16 miRNAs were differentially expressed in a cohort of 13 subjects with the disorder and 21 psychiatrically unaffected controls [76]. Our group initially investigated miRNA expression in postmortem superior temporal gyrus (STG) from 21 subjects with the disorder and 21 non-psychiatric controls and observed the up regulation of the brain-enriched miRNA, miR-181b. This was accompanied by the downregulation of miR-181b target genes with an implication in schizophrenia development, including VSNL1, a member of the visinin/recoverin subfamily of neuronal calcium sensor proteins, and the glutamate receptor GRIA2, which is involved in synaptic plasticity [77]. After further analysis and the addition of the dorsolateral prefrontal cortex (BA9), we revealed that a larger proportion of miRNA were elevated, suggesting a global increase in miRNA biogenesis. This hypothesis was supported by an increase in the microprocessor component DGCR8 and miRNA maturation enzyme Dicer1. An interesting feature of the altered miRNA expression in both of these regions was the over-representation of miR-107 and members of the miR-15 family, which have similar miRNA seed regions, and common target genes are involved in axon guidance, long-term potentiation, Wnt signaling and Mitogen-activated protein (MAP) kinase signaling. Many of these target genes, such as RELN, DRD1, VSNL1, and HTR2A, with known function in neural connectivity, have already been associated with the development of schizophrenia [78].

We also investigated miRNA expression in a larger cohort of 37 matched pairs of case/control subjects in postmortem DLPFC (BA46) and observed 25 upregulated and 3 downregulated molecules in subjects with schizophrenia, many of which were brain-enriched and showed neuron-specific expression. Again, the overall increase in miRNA expression was supported by an increase in the expression of the miRNA biogenesis genes DICER1 and DGCR8. Pathway analysis for these genes indicated their enrichment in axon guidance and long-term potentiation pathways, which are both significant to schizophrenia. RT-qPCR confirmed the differential expression of six of these miRNAs, including miR-328, miR-17-5p, miR-134, miR-652, miR-382, and miR-107 [79]. More recently we integrated the differentially expressed miRNA with mRNA from the same tissue and observed substantial correlation networks between miR-92a, miR-495, and miR-134, and their target genes in pathways involved in neurodevelopment and oligodendrocyte function. This was directly supported in reporter gene assay for interactions with BCL11A, PLP1, and SYT11 [80].

In another postmortem study of the DLPFC in schizophrenia, miR-132 was observed to be dysregulated and this was supported by the altered expression of its target genes, including DNMT3A, GATA2, and DPYSL3, which are known to be related to the development of the nervous system and schizophrenia [72]. Kim et al. had previously reported the downregulation of miR-132, alongside six other miRNAs, including miR-132-3p, miR-212, miR-544, miR-34a, miR-7, and miR-154-3p, in the prefrontal cortex of individuals with schizophrenia [81]. Consistent with the assumption that schizophrenia development is influenced by gender and probably regulated by estrogen signaling, a study showed that the expression of the estrogen-sensitive miRNA, miR30b, is significantly decreased in the cerebral cortexes of female, but not male, patients [82]. miR-17 is a neurodevelopment-associated miRNA whose upregulation was reported in the prefrontal cortex of schizophrenia subjects, accompanied by the decreased expression of its experimentally validated target, NPAS3, in a subpopulation of patients. NPAS3 is a transcription factor that is important for development and associated with psychotic illness [83].

In a recent study exploring postmortem amygdala from 13 schizophrenia cases and 14 controls, Liu et al. reported the significant upregulation of miR-34a as well as the three-fold downregulation of a novel miRNA, miR-1307 [84]. Interestingly, the locus encompassing miR-1307 was highly significant in the PGC schizophrenia GWAS (rs11191419, *p* value = 6.198 × 10^−19^). The details of discussed miRNA studies are summarized in Table 2. Collectively, there are now substantial data, suggesting that miRNAs are dysregulated in schizophrenia and, in many cases, the networks they regulate are enriched with target genes with synaptic function that, if disrupted, could adversely affect neural connectivity during the development of the disorder.

### 3.3. Long Non-Coding RNAs (lncRNAs)

Long non-coding RNAs (lncRNAs) are non-protein coding transcripts of greater than 200 nucleotides in length. Structurally, lncRNA genes share many features with protein-coding genes, such as having promoter regions and exon–intron boundaries. Their transcripts also have common characteristics with mRNAs, such as carrying a 5′ cap and a 3′ poly-A tail, as well as undergoing alternative splicing and RNA editing. Their main difference, as the name implies, is that lncRNAs, with very few exceptions, are not translated [96]. Genes encoding lncRNAs are spread across genomes, and their relative location forms the basis of their classification. Examples include long intergenic RNAs (lincRNAs), which are transcribed from genes located between protein-coding regions of the genome, and antisense lncRNAs (asRNAs), originated from antisense strands of protein-coding genes. Functionally, lncRNAs can regulate gene expression at the post-transcription level by functioning as competing endogenous RNA (ceRNA), particularly for miRNA [97]. Second, as shown in the case of the antisense lncRNA BACE-1 and miR-485-5p [98], lncRNAs can prevent the miRNA-mediated repression of translation through competing with miRNAs for binding to their target sequence (MRE) on mRNA molecules, and therefore increasing their stability. Third, another form of this so-called sponge activity of lncRNAs was observed by Wang et al. in human embryonic stem cells [99]. They reported that the lincRNA linc-ROR shares with the transcription factors OCT4, NANOG and SOX2 their miRNA response elements, and so, can function as a competing endogenous RNA (ceRNA) to sponge miRNAs, suppressing their repressive effect on TF activity [99] (Figure 1). Conversely, lncRNAs often have miRNAs embedded in their sequence and host miRNA precursors to control their synthesis and release. miRNAs also are able to target lncRNAs and regulated their abundance by mediating their degradation [100].

There are also several mechanisms that lncRNAs can use to modify expression even earlier at the level of transcription. Firstly, they can alter chromatin states by guiding chromatin modifying complexes to specific sites of activity in the genome [101] (Figure 1). For example, Tsai et al. showed that the lincRNA HOTAIR binds to two histone modification complexes, polycomb repressive complex 2 (PRC2) and LSD1/CoREST/REST, through its 5′ and 3′ domains, respectively, to coordinate their respective histone H3 lysine 27 methylation and lysine 4 demethylation activities at target sites [102]. Khalil et al. have previously shown that the depletion of specific PRC2-associated lincRNAs resulted in gene expression upregulation, especially for those normally suppressed by PRC2, indicating the vital role of lincRNAs for PRC2 functionality (Figure 1) [103]. LncRNAs can also form associations with transcription factors and direct their localization at target genes [104]. For instance, the brain-specific lncRNA RMST interacts with transcription factor SOX2 and together they regulate the transcription of a large number of genes. Intriguingly, it has been shown that it is RMST, not SOX2, which binds to target genes promoters [105].

### 3.4. LncRNA Studies in Postmortem Brain

The earliest evidence of lncRNA involvement in schizophrenia pathology emerged from a linkage study on a Scottish family which showed the association of a translocation with various mental disorders, such as schizophrenia, bipolar disorder, and major depression. The region encompassed the protein-coding gene DISC1 and its human-specific anti-sense transcript DISC2, which was then assumed to act as a natural antisense transcript (NAT) to suppress DISC1 expression [106,107]. More recently investigators have again turned to postmortem brain to explore the expression of these molecules.

Barry et al. showed that the brain-enriched long non-coding RNA Gomafu, also known as MIAT and RNCR2, interacts with several splicing factor proteins, such as SF1 and QKI, and takes part in the alternative splicing of DISC1 and ERBB4, both involved in schizophrenia etiology. It was significantly downregulated in response to depolarization in mouse primary neurons and human-induced pluripotent stem cells (hiPSCs). In addition, they reported a 1.75-fold reduction in Gomafu expression in the STG of 28 schizophrenia subjects relative to 28 controls [108]. Later, the SNP rs1894720, proximal to Gomafu, was reported to be significantly associated with paranoid schizophrenia in a discovery sample of 1093 cases and 1180 controls, as well as a replication cohort of 1255 cases and 1209 controls, from a Han Chinese cohort [109]. Following this study, knock-down and the resultant down regulation of Gomafu in a mouse peripheral cortex was shown to result in anxiety-like behavior [110]. These observations, alongside the fact that the chromosomal locus encompassing Gomafu, 22q12.1, is suggested to be linked with schizophrenia-associated eye movement disorder, further support Gomafu’s involvement in the pathophysiology of schizophrenia [108].

Applying RNA-sequencing on postmortem brain samples, Hu et al. identified the differential expression of 35 lncRNAs in two brain regions—the dorsolateral prefrontal cortex (BA9) and anterior cingulate cortex (BA24). Some of the detected lncRNAs were related to neuron ensheathment, metabolic processes, myelination, and oligodendrocyte differentiation [111]. More than 200 lncRNAs were also reported to be differentially expressed in postmortem amygdala from 22 individuals with schizophrenia compared to 24 control subjects, three of which, AC005009.2, RP11-724N1.1, and RP11-677M14.2, were located in schizophrenia-associated loci [84].

In recent work, we observed a significant depletion of covalently closed circular lncRNA (circRNA) in postmortem DLPFC (BA46) in schizophrenia [112]. These molecules were enriched in the brain, have high stability and high capacity for miRNA binding, particularly those previously shown to be associated with schizophrenia. We suspect this reduced capacity to sponge miRNA may further exacerbate the functional consequences of increased miRNA expression observed in the same tissue [79].

More generally, lncRNAs have been shown to respond to many environmental stressors, such as oxidative stress, genotoxic agents and heavy metals, so it is plausible that some of these signals encoded by these molecules contribute to the neuropathology of schizophrenia [113]. In future work it will be interesting to further explore the link between environmental exposures and changes in lncRNA expression with functional significance for brain development and connectivity.

## 4. Schizophrenia-Associated Epigenomic Changes in Peripheral Tissues

Given that the common environment effects that induce changes in gene expression in the brain are also experienced by the entire body, it is highly plausible that many of the epigenomic marks left by this exposure will also manifest in peripheral tissues. This is supported at the transcriptional level with similarities between whole blood and multiple CNS tissues, including about half of a set of schizophrenia candidate genes [114], suggesting that many of the epigenetic mechanisms may be conserved between tissues. For example, the S-COMT promoter was observed to have similar methylation pattern in the blood and brain [115]. A recent transcriptome-wide mega analysis also reported the joint dysregulation of immunologic genes and transcription regulators in the blood and brains of individuals with schizophrenia [116]. Auta et al. showed the comparability of the expression increase in TET1 and DNMT1, two DNA methylation/demethylation enzymes, as well as BDNF and GCortR, two schizophrenia candidate genes, in the peripheral blood lymphocytes (PBL) of schizophrenia patients with those observed in the postmortem brain [117]. These observations have led to a growing interest in epigenetic studies of blood as an easily accessible tissue from living patients, to determine both schizophrenia etiology and biomarkers. Other peripheral tissues, such as olfactory and buccal epithelium, as well as extracellular fluids, such as serum, saliva, and cerebrospinal fluid, have also been explored in relation to schizophrenia and provide an alternative view of the state of the disorder.

### 4.1. DNA Methylation in the Periphery

According to Aberg et al. [118,119], methylation changes associated with disease-related events are conserved and detectable in the blood. This is called the “signature model”. However, they also propose an alternative view named the “mirror-site” model, where the methylation status of a site in the blood reflects the methylation status of a corresponding site in the brain that may be of relevance for the disease’s etiology. In accordance with this model, Walton et al. reported that a proportion of peripheral changes in methylation may act as a proxy for brain tissue methylation status; however, they emphasize that most DNA methylation markers in peripheral blood do not reliably predict brain DNA methylation status [120]. Very recently, a genome-wide methylation study on blood, saliva, and brain tissue, collected during neurosurgical intervention from 27 individuals with epilepsy, revealed relatively high levels of correlation between saliva and brain (*r* = 0.9) and between blood and brain (*r* = 0.86). Intriguingly, while the across-subject analysis showed a high degree of similarity among tissues, within-subject results indicated that most CpGs had dissimilar patterns [121]. Here, we generalize the signature model to other forms of epigenetic changes, including histone modifications, miRNAs and lncRNAs, and assume that the environmental exposures, which dispose individuals to developing schizophrenia, leave signatures in the peripheral tissues and, therefore, these types of tissues can be investigated not only for finding biomarkers, but also with the aim of unravelling disease etiology.

Some concordance for methylation status has been observed between blood and brain in relation to schizophrenia candidate genes, including RELN and COMT. The RELN promoter was observed to be hypermethylated in the peripheral blood of people with the disorder compared to the unaffected controls [21], which was consistent with postmortem methylation in the brain [28,29]. S-COMT hypermethylation was also observed in the leukocytes of schizophrenia patients (*n* = 177) compared to controls (*n* = 171) [122]. While this was later confirmed in another study, the results were not observed in females, suggesting that this influence is sexually dimorphic [123]. Interestingly, another study by Nohesara et al., looking at saliva as a convenient peripheral biomarker for schizophrenia, observed the hypomethylation of MB-COMT [124], which is consistent with brain studies [38] and provides a confirmation for the mirror-site model. We can, therefore, speculate that one contributing factor to schizophrenia development might be the hypermethylation of RELN (involved in axonic and dendritic extension, and neuronal migration), which reduces its expression in the brain, whereas the hypomethylation of the MB-COMT gene may increase the level of its product in the brain and stimulate positive symptoms.

Other observations on candidate genes include hyper-methylation of the glutamate receptors GRM2 and GRM5 in the peripheral blood of schizophrenia subjects [125], hyper-methylation of BDNF promoter in the whole blood of patients [126], promoter hyper-methylation of the serotonin receptor 5HTR1A in peripheral leukocytes [127], and hypo-methylation of the serotonin receptor HTR2A promoter in patient saliva [128], which is compatible with the result of the brain study [129]. Details of the cited methylation studies in candidate genes are summarized in Table 3.

As an alternative to candidate gene-based analysis of differential methylation, some studies have investigated total DNA methylation across the entire genome as a single measure and used this to examine changes related to schizophrenia. While an early study did not reveal global DNA methylation changes in the peripheral blood leukocytes of individuals with schizophrenia compared to healthy controls [131], more recent studies suggest there is global DNA hypomethylation in the peripheral blood of schizophrenia patients with first-episode schizophrenia [42,122] and in discordant twins with the disorder [132]. This was supported more recently with the observation of the global hypo-methylation of peripheral leukocytes in individuals with schizophrenia [133]. While this is interesting, it is far more important to know how these differences are distributed across the genome and determine the consensus genes and pathways that are potentially influenced. To achieve this outcome, researchers have been implementing epigenome-wide association analyses on larger cohorts of blood derived cells using DNA methylation array.

For example, a large EWAS was performed on a buffy coat of whole blood of 759 and 738 Swedish cases and controls, respectively. The top EWAS finding, remaining significant after applying a very conservative correction for multiple testing, was located at gene FAM63B. FAM63B is part of four networks regulated by three miRNAs (miR-218, miR-9, and miR-504) that can be associated to dopaminergic gene expression and neuronal differentiation [119]. Interestingly, a follow-up study on blood samples from bipolar disorder patients replicated FAM63B hypomethylation compared to healthy controls, confirming the possible contribution of this gene to psychiatric illness [134]. More recently, a larger EWAS of schizophrenia consisting of 1714 participants identified 25 differentially methylated positions (DMPs) associated with SZ with a *p*-value of less than 10-7, and this number increased to 1223 DMPs with a more relaxed discovery threshold of *p*-value < 5 × 10^−5^ [135]. The samples were divided into a discovery set derived from the blood of 353 schizophrenia cases and 322 non-psychiatric controls, with the remainder of the cases and controls being used to validate the association. The 1223 DMPs were demonstrated to be significantly enriched in binding motifs of specific transcription factors, including BATF, BCL11A, IRF4 and MEF2A. The 955 genes annotated to these DMPs were enriched in 153 groups of related GO categories, with the first and second groups of pathways related to immune function and neuronal proliferation and brain development, respectively. The researchers also applied two different approaches to identify schizophrenia-associated differentially methylated regions (DMRs). The first approach, comb-p algorithm, returned 12 regions, with the top one spanning 20 CpG sites and overlapping the major histocompatibility complex (MHC), which is the most associated locus in schizophrenia GWAS [5]. The second approach, sliding window, identified 531 regions associated with schizophrenia, constituting 76 non-overlapping regions, each containing 2–120 probes. The top DMR (*p* = 1.87 × 10^−14^) spanned three probes within the gene GYG1, which was previously reported as differentially expressed in prefrontal pyramidal neurons from schizophrenia patients [136], leaving GYG1 as another candidate gene for schizophrenia neuropathological investigation.

Given the broad distribution of differentially methylated CpGs in the blood of individuals, Watkeys et al. hypothesized it would be possible to score the epigenome-wide burden of methylation related to the syndrome in individuals with the disorder using the summary statistics from the aforementioned discovery sample as a reference [137]. The metric for this analysis designated that the poly-methylomic risk score (PMRS) is analogous to the polygenic risk scoring approach used for the common sequence variants captured by GWAS. The predictive utility of this metric was tested in a small validation sample of subjects with schizophrenia and bipolar disorder.

### 4.2. Histone Modification Studies

Reviewing the literature for histone modification studies in peripheral tissues in schizophrenia returned only one result—a pilot study, in which ChiP-seq and microarray were applied to explore genome-wide H3K4me3/H3K27me3 and gene expression, respectively, in the olfactory cells of four schizophrenia patients compared to four controls. The authors detected 22 genes for which schizophrenia-associated changes in expression were likely to be linked with changes in histone trimethylation. qPCR validated expression changes for three genes, MGST1, DAAM2, and LPXN [138]. DAAM2, an actin assembly factor, was previously suggested as a biomarker for schizophrenia diagnosis, as it showed increased expression in the whole blood of the patients, and also as a disease state marker, since its expression returned to baseline levels after complete clinical remission [139].

It is worth mentioning that olfactory epithelium (OE) has recently been explored for the epigenomic evaluation of several brain disorders, including schizophrenia. There are several reasons to consider these cells as a valuable tissue for identifying reliable biomarkers. Firstly, they are an accessible neuronal lineage at multiple stages of maturity, which provide an excellent model for investigating neural development [140,141,142]. Secondly, deficits in olfaction are known to occur in several neuropsychiatric conditions and are likely to be associated with cellular or molecular dysregulation in OE. In schizophrenia, olfactory dysfunction has been associated with negative and cognitive symptoms.

## 5. Schizophrenia and Non-Coding RNA in Peripheral Tissues

### 5.1. miRNA

With such a substantial change in miRNA expression observed in the central nervous system in schizophrenia, it is plausible that similar changes take place in peripheral tissue and may be accessible biomarkers of related disease processes. This hypothesis was supported by the work of Lai et al. in their report of a seven-miRNA signature, including miR-34a, miR-449a, miR-564, miR-432, miR-548d, miR-572, and miR-652 in peripheral blood mononuclear cells (PBMCs) in a small training set of 30 cases and 30 controls and a larger validation set of 60 cases and 30 controls [85]. Interestingly, the most differentially expressed miRNA in both arms of the study was miR-34a, which was consistent with a previous report in the prefrontal cortex [81]. Later, a meta-analysis, coupled with RT-qPCR on PBMCs, validated the applicability of miR-34a, as well as miR-181b-5p, miR-21-5p, miR-195-5p, miR-137, miR-346, and miR-34a-5p, as biomarkers with high diagnostic sensitivity and specificity [86]. Given that the target genes of miR-34a are involved in neurogenesis and neuron differentiation, the disruption of its expression was suggested to be a contributing factor to schizophrenia development.

Recently, a combination of miR-22-3p, miR-92a-3p, and miR-137 was suggested as a schizophrenia diagnosis biomarker following an investigation on peripheral blood from ten patients and ten healthy controls [91]. Yu and collaborators applied microarray and qRT-PCR to investigate for disease biomarkers in the PBMCs of 105 schizophrenia patients compared to 130 psychiatrically healthy people and observed a decreased expression of miR-132 [95]. Intriguingly, this miRNA had previously shown to be down-regulated in the prefrontal cortex of schizophrenia cases [72], providing more evidence for the mirror-site model and potential utility of peripheral tissues for schizophrenia etiology studies.

In our laboratory, PBMCs from 112 schizophrenia patients and 78 controls were examined and we identified the downregulation of 34 microRNAs at FDR = 0, three of which, including miR-134, miR-128 and miR-181b, are brain enriched. The differential expression of seven of these microRNAs, including miR-31, miR-431, miR-433, miR-107, miR-134, miR-99b, and miR-487b, was further validated by qRT-PCR in a representative sample of 57 cases and 34 controls. Interestingly, of the most significantly downregulated miRNAs, 17, including miR-134, are clustered in two closely neighboring positions on chromosome 14; at 14q32.2 and 14q32.31. Notably, this locus, known as the DLK1-DIO3 region, is imprinted such that the associated miRNA cluster is only expressed from the maternal chromosome. This, alongside the observation that no CNV had occurred in this region in a subset of samples including 57 controls and 81 cases, implicated the plausible epigenetic regulation of this cluster expression [87]. Moreover, considering that the differential expression of miR-134 was also observed in our brain study, although in the reverse direction [79], we proposed this miRNA as another candidate gene with significance for the pathophysiology of schizophrenia. In a similar study, we recently applied RNA-sequencing to investigate miRNA expression in PBMCs from 36 schizophrenia cases and 15 controls, and demonstrated the differential expression of 35 miRNAs, three of which were further validated by qRT-PCR; miR-1271-5p, miR-221-5p, which is an immune response activator, and let-7f-5p, which is involved in neurodevelopment [92].

The differential expression of miRNAs has also been reported in the plasma of schizophrenia patients. A cohort of 17 individuals with first episode schizophrenia and 17 healthy controls was examined by Zhao et al. using microarray analysis, who observed the significant elevated expression of miR-223 and validated it by quantitative reverse transcription-polymerase chain reaction (qRT-PCR) in another independent sample of 21 patients and 21 controls. Interestingly, this miRNA was previously shown by our group to be up-regulated in the dorsolateral prefrontal cortex of schizophrenia patients [78]. The authors experimentally confirmed the direct interaction of miR-223 with four target genes, INPP5B, RHOB, SKIL, and SYNE1, which are all related to neural development and neuronal migration and, therefore, hypothesized that miR-223 can contribute to the etiology of schizophrenia [93].

More recently, Liu et al. hypothesized that a transcription factor-miRNA-target gene axis might serve better as a biomarker than a single miRNA [143]. Working on PBMCs from a cohort of 38 schizophrenia patients and 50 healthy controls, they focused on miR-30 members, as the dysregulation of this family has been shown previously in several studies [76,82,87]. Among three transcription factors, which regulate the expression of miR-30 family members, only EGR1, with downregulation reported in postmortem prefrontal cortex (PFC) in schizophrenia [144], had significantly lower mRNA level in PBMCs of patients. Of three EGR1-regulated miRNAs, the levels of miR-30a-5p and miR-30e-5p were found to be significantly lower in patients. As the only target of miR-30e-5p, UBE21, does not have any reported role in schizophrenia, further studies were focused on schizophrenia-associated target genes of miR-30a-5p—i.e., BDNF, SMAD1, and NEUROD1, among which only NEUROD1 showed a significantly higher expression in patients. The alteration of the EGR1-miR-30a-5p-NEUROD1 axis was then validated by RT-PCR in patient’s PBMCs, and further analyses confirmed the significantly higher diagnostic value of this axis compared to miR-30a-5p by itself.

While early investigations focused on exploring cellular RNA molecules from the CNS and peripheral tissues, miRNA can also survive quite well in extracellular fluid and are protected from nuclease decay by Argonaute protein or through encapsulation in extracellular vesicles. miRNAs that are packed in cellular secretory vesicles or ‘exosomes’ are thought to be able to regulate recipient cell function following the fusion of exosomes to their plasma membrane. Interestingly, Banigan et al. explored exosomal microRNAs from postmortem PFC and observed an increased expression of miR-497 in schizophrenia subjects [88]. Serum miRNAs, referred to as circulating miRNAs, have also been observed to be altered in the disorder. Although the functional significance of serum miRNA is not yet known, they are already under investigation as biomarkers of several disease conditions. For example, two studies in 2008 reported the application of specific serum miRNAs for distinguishing patients with non-small cell lung cancer and prostate cancer from healthy controls, and showed that they are highly stable under extreme conditions, such as ribonuclease digestion and extreme pH and temperature [145,146]. While the origin of circulating miRNAs also remains unclear, it has been reported that they may emerge from three different mechanisms, including passive leakage from broken cells, active secretion as microvesicles, and active secretion through an RNA-binding protein-dependent pathway. However, it has been suggested that active secretion of microvesicles is the major source of circulating miRNAs [147,148].

In the first study on serum miRNAs expression in schizophrenia [89], with 115 patients and 40 controls, seven molecules were identified as potential biomarkers, including miR181b, whose dysregulation in superior temporal gyrus [77] and PBMCs [87] had been previously reported by our group. More recently, circulating miRNAs were profiled in a test cohort of 164 schizophrenia patients and 187 control subjects, using sequencing, microarray, and qPCR assays. The captured miRNAs were then validated by qPCR in an independent cohort of 400 schizophrenia patients, 213 control subjects, and 162 patients with non-schizophrenia psychiatric disorders. Plasma miRNA screening identified eight upregulated miRNAs in schizophrenia, with qPCR analysis supporting the upregulation of miR-130b and miR-193a-3p in schizophrenia but not in other disorders tested, suggesting that these two miRNAs could be used to develop a diagnostic tool for schizophrenia [90]. Interestingly, miR-130b is expressed in the brain, located in a susceptibility locus for schizophrenia (22q11), and potentially targets MECP2, which is involved in the normal development of the brain and was considered a plausible candidate for involvement in schizophrenia [149].

More recently, small RNA-sequencing was applied to blood exosomes from a cohort of 49 schizophrenia cases and 46 controls and identified significant expression changes in 26 miRNAs, six of which had more than a 2-fold change in patients compared with the controls, including miR-145-5p, miR-144-5p, miR-206, miR-133a-3p, miR-184, and miR-144-3p. The observed up-regulation of miR-206 was accompanied by the down-regulation of its target, BDNF, in the blood. Since decreased expression of BDNF has been negatively associated with cognitive functions of animals, the researchers concluded that exosomal hsa-miR-206 up-regulation may contribute to BDNF dysfunction in schizophrenia, a finding which further supports the neurotrophin hypothesis of schizophrenia [94]. The details of miRNA studies covered in this section are summarized in Table 2.

One important point to take into account regarding serum biomarkers is that most of the miRNA detectable in body fluids are expressed in many tissues, and a large proportion are thought to originate from blood cells, such that disturbances in blood cell counts and hemolysis can change plasma levels of miRNA biomarkers by up to 50-fold [150]; so, the observation of alterations in their concentrations in one pathology does not mean that the same miRNA cannot contribute to other diseases. So, it is crucial for the candidate biomarkers to be tested in other pathologies as well, not only in healthy control subjects [151].

### 5.2. lncRNA

While aberrant lncRNA have recently been implicated in the neuropathology of schizophrenia, changes in their peripheral expression may also serve as state and trait biomarkers for the disorder. Ren et al. examined PBMCs from a cohort consisting of 19 first-episode early-onset schizophrenia (EOS) patients and 18 controls. Instead of differential expression analysis at the level of individual genes, they applied weighted gene co-expression network analysis (WGCNA) to identify higher order relationships among gene products. Their analysis revealed two schizophrenia-associated lncRNA modules. They did the same analysis for mRNA which returned three disease-associated mRNA modules and then combined the two datasets through canonical correlation analysis (CCA) and observed the disturbance of mitochondria, which is consistent with other studies, indicating the role of mitochondrial dysfunction in schizophrenia development [152].

Differential expression of lncRNAs in PBMCs was also reported by Chen et al. [153]. Applying a lncRNA oriented microarray technique, they first showed significant differential expression of 125 lncRNAs, 62 upregulated and 63 downregulated, in a discovery sample of three schizophrenia subjects and three healthy controls, five of which were further validated by qRT-PCR in a cohort of 106 cases and 48 controls, namely NONHSAT089447, NONHSAT021545, NONHSAT041499, NONHSAT098126, and NONHSAT104778. qRT-PCR also revealed the downregulation of NONHSAT089447 and NONHSAT041499, concordant with the decrease in PANSS (Positive And Negative Syndrome Scale (PANSS) scores, following patient treatment with antipsychotic medication. Based on these observations the authors suggested the involvement of the two lncRNAs in schizophrenia pathogenesis and development.

## 6. Discussion and Perspectives

In this review we have outlined some of the research exploring the epigenomic landscape of schizophrenia. For obvious reasons, a large proportion of this has focused on the brain, where changes in chromatin structure or the dynamics of post-transcriptional regulation have a clear impact on etiological roots, the current state, and future course of illness. These changes, in many cases, are the legacy of many years of the condition and reflect the impact of both heritable components and environmental exposures. The biological impact of these modifications on the expression of genes is likely to alter the fine balance of molecular networks regulating neural connectivity and is consistent with the neurodevelopmental hypothesis of schizophrenia. Significantly, where these changes can be ascertained in individuals, there is an opportunity for personalized interventions that can tap into the network dynamics and make adjustments that are specific to an individual’s genetic and epigenetic status.

To achieve this kind of outcome in precision medicine, we need to be able to determine minimally invasive biomarkers of epigenomic changes in tissues and biological fluids that are reflective of states that prevail in the brain and affect behaviors that are relevant to psychiatric disorders. In view of this, we also examined research that is attempting to identify peripheral biomarkers of epigenomic changes in schizophrenia and found that, in some cases, these accord with those changes that take place in the central nervous system. This is perhaps not surprising, given that the entire body shares both its genetic instructions and most of the common environmental exposures that are associated with psychiatry disorders. Even those exposures, such as psychosocial stress, that we typically associate with the brain, have a very significant impact on the entire body through the systemic circulation of glucocorticoids, cytokines, and a myriad of other messenger molecules, including non-coding RNA.

**Figure 2:**
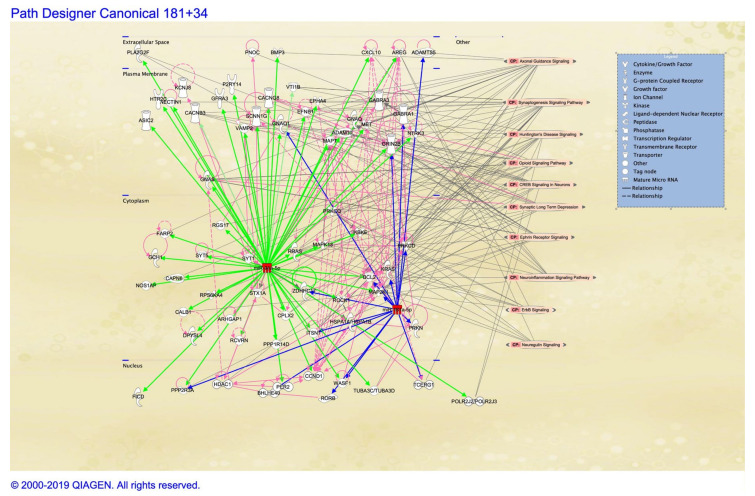
Putative target gene interaction network graph generated using Ingenuity Pathway Analysis of miR-34 and miR-181 family miRNAs nodes within a simulated cell. Target mRNA interactions are denoted by green and blue edges for the seed sequences GGCAGUG and ACAUUCA using high stringency target predictions (TargetScan) and experimental observation for miR-34 and miR-181, respectively. Protein–protein interactions between miRNA targets are illustrated in pink. Gene ontologies related to neural functions are projected from the right side (grey lines) with the highest number of interactions at the top (axonal guidance signaling) and the least at the bottom (neuregulin signaling).

The case for concordance between the brain and peripheral tissue was exemplified by miR-181b, with its observed upregulation in the brain (STG) [77] and serum [86,89]. It has also been shown to be downregulated in PBMCs [87]. Similarly, so does miR-34a, with studies indicating its upregulation in amygdala [84] and PBMCs [85,86], and downregulation in PFC [81]. In Figure 2 we explore the network of putative interaction between these microRNA in respect to neurobiologically significant ontologies. While histone and DNA modification in the candidate genes RELN, GAD1, COMT, and SOX10 are also exemplars of brain body concordance, in respect to chromatin, these probably only represent the tip of the iceberg, reflecting only the bias of the hypothesis derived from our preconceptions of the underlying biology. Newer high-throughput approaches that enable the exploration of genome-wide changes in the epigenomic footprint, both through sequencing and high-content microarray are providing the prospect of much greater refinement to our models and improved predictive capability. As these mature, we should be able to identify the changes that reliably capture the phenotypic variance for schizophrenia, particularly the components not already accounted for by polygenic risk, and use this to better understand the individual (in the context of population heterogeneity) to inform treatment.

While biomarkers often strive to mimic diagnostic classification to support early intervention, they can also be used to subgroup affected individuals with the disorder into biotypes that may reflect a shared genomic architecture or exposure that drives the etiology. Some of the biomarker research, including that derived from epigenomic features, therefore, focuses on disease endophenotypes. A number of the epigenomic changes described in this review have drawn support from this type of study. For example, exploring the treatment resistance endophenotype of schizophrenia, Alacam et al. tested 29 disease-associated miRNAs and observed that miR-181b-5p, as well as two others, were differentially expressed in 18 treatment-resistant schizophrenia patients compared to 19 responders. They observed that the level of miR-181b increased approximately three-fold in the treatment resistant group compared to healthy individuals and decreased by 0.76-times in the treatment-responsive group. The expression of miR-181b-5p also decreased in the responsive group during the treatment, supporting the previous observations [154], suggesting this miRNA is associated with treatment resistance and disease etiology [155]. Similarly, epigenomic changes in the schizophrenia candidate gene RELN is also supported by its relationship with the cognitive function of schizophrenia patients. This was evidenced by the hypomethylation of CpGs in a specific region of the RELN promoter region, which was associated with the development of cognitive deficits in schizophrenia [156].

The up-regulation of miR-137 in peripheral tissues in schizophrenia was also reported in three independent studies, suggesting it may have applications as a biomarker [86,91,157]. miR-137, first identified in the mouse midbrain cortical tissue [158], is a brain-enriched miRNA predicted to target over 1000 genes involved in various pathways, such as cell cycle, neural development, proliferation, and differentiation. Experimental evidence supports the involvement of miR-137 in the regulation of adult neurogenesis, dendritic development, and neuronal maturation [159]. Its involvement in schizophrenia was supported by the genome-wide association of almost 37,000 cases and 113,000 controls, with the second most associated variant in proximity to the MIR137 gene (rs1702294, *p* = 3.4 × 10^−19^) [5]. Despite strong association, the dysregulation of miR-137 expression has not been reported in postmortem studies of the disorder, even when taking the genotype into account, whereas the effect of the variant on gene expression has been observed in controls [160]. Interestingly the variant has been associated with cognitive deficit in schizophrenia [161], so it is plausible that the cortical expression of this miRNA is indicative of a more clinically challenging presentation of this disorder.

While the search for clinically useful diagnostic biomarkers for neuropsychiatric disorders has a history spanning several decades, there is little in the way of successful outcomes to report for several reasons. Perhaps the most important of these is the existence of significant levels of heterogeneity which, in some cases, can be reduced to different subtypes for many of these disorders, which is reflected in the high levels of variation in gene expression or chromatin modification between tissues and patient populations [162]. While there has been great progress towards covering the common variant genetic heterogeneity through large mega GWAS and meta-analysis there have been no comprehensive meta-analyses on epigenetic diagnostic biomarker studies. Some of the non-epigenetic diagnostic biomarkers suggested for SZ, which have received support from meta-analyses, include decreased levels of pyridoxal (Vitamin B6) and nerve growth factor (NGF), and soluble interleukin-(IL)-2 receptor (sIL-2R) level increase, confirmed by a recent umbrella review [163], as well as a significantly increased level of the acute-phase protein C-Reactive Protein (CRP) [164]. On the other hand, pinpointing prognostic biomarkers, that can predict the future risk of developing schizophrenia before the onset of disease symptoms, is of critical importance. This is not convenient and time-efficient in human studies and, therefore, developmental animal models are required, through which the effects of perinatal and/or early postnatal exposure to environmental manipulations and/or drug administration are investigated in peripheral tissues over the course of development [165]. Our literature search for these types of studies returned only one result, in which pregnant mice were treated with the toxicant bisphenol A (BPA), that disturbs neurodevelopment with long-term effects on behavior, and the offspring went through gene expression and DNA methylation analyses in the hippocampus and blood. Based on the results, the authors suggested that BDNF DNA methylation in the blood can predict early life adversity-induced epigenetic changes in the brain and may be a novel clinical epigenetic biomarker to predict the risk for psychopathology [166].

To conclude, epigenomic features and associated non-coding RNA provide an important mechanism for the neuropathology of schizophrenia that, in some cases, can be tracked through peripheral tissues. Where these provide important information/biomarkers that inform on an individual’s trait and state risk, they may have utility to both facilitate early intervention and direct intervention in support of precision medicine strategies. Future work that better captures and integrates these forms of information through poly-omic methodologies will make an important contribution toward this aim by providing the training sets needed to establish an unbiased etiological framework. While much of this exploration is focused specifically on disease, more studies using alternative phenotypic labels related to sub-types or other endophenotypes will further refine our models and provide the basis for more cross-disorder integration of psychiatric and behavioral syndromes.

## Figures and Tables

**Table 1:** DNA methylation studies in postmortem brain.

Cohort Size	Brain Region	Approach	Main Findings	Gene(s)	Reference
5 SZ/5 Con (all males)	Frontal lobe	Bisulfite sequencing and methylation-specific PCR (MSP)	Hyper-methylation of RELN promoter in SZ	RELN	[28]
15 SZ/15 Con	Occipital and prefrontal cortices	Bisulfite sequencing and nested PCR	Hyper-methylation of RELN promoter in SZ	RELN	[29]
14 SZ/13 Con	Prefrontal cortex	Pyrosequencing	No detectable differences in DNA methylation of RELN	-	[30]
35 SZ/35 Con	Frontal cortex	Bisulfite sequencing	No detectable differences in DNA methylation of RELN and COMT/Hypomethylation of NR3B and GRIA2	NR3B and GRIA2	[31]
40 SZ/40 Con	DLPFC (Broadman’s area 46)	Bisulfite sequencing and MSP	Hypomethylation of MB-COMT promoter	MB-COMT	[38]
11 SZ/12 Con	Prefrontal cortex (BA10)	Bisulfite sequencing	Hyper-methylation of SOX10 in SZ	SOX10	[39]
13 SZ/12 Con	STG and parahippocampus gyrus	Bisulfite sequencing	Hyper-methylation of FOXP2 in SZ	FOXP2	[37]

**Table 2:** miRNA dysregulation in schizophrenia.

Cohort Size	Tissue	Technique	Upregulated miRNAs	Downregulated miRNAs	Reference
13 SZ/21 Con	PFC	Microarray and qRT-PCR	miR-106b	miR-26b, miR-30b, miR-29b, miR-195, miR-92, miR-30a-5p, miR-30d, miR-20b, miR-29c, miR-29a, miR-212, miR-7, miR-24, miR-30e, miR-9-3p	[76]
21 SZ/21 Con	STG	Microarray and qRT-PCR	hsa-let-7g, miR-181b	-	[77]
15 SZ/15 Con	STG and DLPFC	Microarray and qRT-PCR	miR-181b, miR-107, miR-15a, miR-15b, miR-195, miR-16, miR-20a, miR-19a, miR-26b, has-let-7e	-	[78]
35 SZ/34 Con	DLPFC	Microarray and qRT-PCR	has-let-7b, miR-15b, miR-32-3p, miR-383, miR-490-5p, miR-196b, miR-513-5p, miR-876-3p, miR-449b, miR-297, miR-188-5p, miR-187	miR-132-3p, miR-132-5p	[72]
35 SZ/35 Con	PFC	Microarray and qRT-PCR	-	miR-34a, miR-132, miR-132-5p, miR-7, miR-212, miR-544, miR-154-3p	[81]
30 SZ/30 Con	PFC and parietal cortex	qRT-PCR	-	miR-30b (only in females)	[82]
37 SZ/37 Con	PFC	Microarray and qRT-PCR	miR-17	-	[83]
13 SZ/14 Con	Amygdala	RNA-Seq	miR-34a	miR-1307	[84]
30 SZ/30 Con	PBMC	qRT-PCR	miR-34a, miR-449a, miR-564, miR-548d, miR-572, miR-652	miR-432	[85]
330 SZ/202 Con	PBMC and Serum	Meta-Analysis	miR-181b-5p, miR-21-5p, miR-195-5p, miR-137, miR-34a	miR-346-5p	[86]
112 SZ/78 Con	PBMC	Microarray and qRT-PCR	-	miR-134, miR-128, miR-181b See the paper for the complete list	[87]
8 SZ/13 Con	PFC Exosomes	Luminex miRNA Assay	miR-497 See the paper for the complete list	See the paper for the complete list	[88]
115 SZ/40 Con	Serum	qRT-PCR	miR-181b, miR-219-2-3p, miR-1308, has-let-7g, miR-346	miR-195	[89]
164 SZ/187 Con	Serum	Sequencing, Microarray and qRT-PCR	miR-122, miR-130a, miR-130b, miR-193-a-3p, miR-193b, miR-502-3p, miR-652, miR-886-5p	-	[90]
10 SZ/10 Con	Peripheral blood	Sequencing and qRT-PCR	miR-22-3p, miR-30d-5p, miR-30e-5p, miR-92a-3p, miR-137, miR-148b-5p, miR-181a-3p, miR-181a-5p, miR-181b-5p, miR-195-5p, miR-199b-5p, and miR-497-5p	-	[91]
36 SZ/15 Con	PBMC	Sequencing	-	let-7f-5p, miR-1271-5p, miR-221-5p	[92]
17 SZ/17 Con	Plasma	Microarray	miR-223	-	[93]
49 SZ/46 Con	Blood exosomes	Sequencing	miR-145-5p, miR-206, miR-133a-3p, miR-184,	miR-144-5p, miR-144-3p	[94]
105 SZ/130 Con	PBMC	Microarray and qRT-PCR	-	miR-132, miR-664-5p, miR-1271, miR-200c, miR-432, miR-134	[95]

**Table 3:** DNA methylation studies in peripheral tissues.

Cohort Size	Tissue	Approach	Main Findings	Gene(s)	Reference
110 SZ/122 Con	Whole blood	Bisulfite modification and qPCR	Hyper-methylation of RELN promoter in SZ, accompanied by RELN expression downregulation	RELN	[21]
177 SZ/171 Con	Leukocytes	Pyrosequencing	Hyper-methylation of S-COMT in SZ patients	S-COMT	[122]
120 SZ/105 Con	Leukocytes	Bisulfite pyrosequencing	COMT hypermethylation in male patients	COMT	[123]
63 SZ/76 Con	Saliva	Bisulfite sequencing and q-MSP	Hypomethylation of MB-COMT promoter in SZ	MB-COMT	[124]
81 SZ/71 Con	Blood	Methylation-specific polymerase chain reaction (MS-PCR)	Hyper-methylation of GRM2 and GRM5 in SZ, accompanied by reduced gene expression	GRM2 and GRM5	[125]
100 SZ/100 Con	Peripheral blood cells (PBC)	Pyrosequencing	Hyper-methylation of BDNF promoter	BDNF	[130]
40 SZ/67 Con	Leukocytes	Bisulfite modification and PCR	Promoter hyper-methylation of 5HTR1A	5HTR1A	[127]
63 SZ/76 Con	Saliva	Bisulfite sequencing and qMSP	Hypomethylation of HTR2A in SZ	HTR2A	[128]
